# MARINS: A Mobile Smartphone AR System for Pathfinding in a Dark Environment

**DOI:** 10.3390/s18103442

**Published:** 2018-10-13

**Authors:** Pei-Huang Diao, Naai-Jung Shih

**Affiliations:** Department of Architecture, National Taiwan University of Science and Technology, 43, Section 4, Keelung Road, Taipei 106, Taiwan; diaoph@msn.cn

**Keywords:** navigation, pathfinding, dark environment, augmented reality, mobile

## Abstract

Traditional egress routes are normally indicated on floor plans and function as designed, assuming that people can identify their relative location and orientation. However, the evacuation process can easily become complicated in a dark or hazardous environment with potential blockage of unexpected obstacles. This study developed the mobile AR indoor navigation system (MARINS) using a smartphone as a device to guide users to exits in a 0-lux setting with the path only illuminated by the phone camera’s LED. The system is developed using Apple ARKit SDK with the associated simultaneous localization and mapping (SLAM) function on a Unity platform in four modules. A maze scenario is planned in an environment built by carton walls. Time and distance traveled by the experimental group and the control group are measured. The results of statistical analysis demonstrate that the MARINS system can reduce travel time in known space and in total summation compared to the application of a traditional map. The system also reduces travel distance and misjudgments with higher system usability than the application of a traditional map.

## 1. Introduction

Each building is organized by a certain kind of circulation system and space hierarchy as a maze of different complexity. To traverse from one location to another in a maze, the knowledge of space comprises a person’s absolute location in a maze, the relative location between the current location and the goal or exit, and the connection in between. While certain path-searching rules may apply or egress routes may possess fail-free designs, each movement necessitates clear visibility of an environment, which can be challenging when power failure occurs to a lighting system. Consequently, it would be greatly beneficial if an indoor navigation system could be developed specifically for a power failure situation using a ubiquitous device, such as a smartphones.

To solve the above-mentioned problems, this study applies simultaneous localization and mapping (SLAM) technology provided by ARKit SDK to develop a simple location-based AR navigation system for a totally dark environment, with a smartphone LED as the only light source. The mobile AR indoor navigation system (MARINS) is applied to a predefined indoor maze in a 0-lux darkness condition. In comparing a 2D map and MARINS, this study aims to determine if a system can be developed to achieve a shorter evacuation time, less misjudgments of distance traveled, and a better system acceptance and usability, based on a statistical analysis of an experimental group and a control group. In addition, a path selection option is provided for unexpected obstruction for known and unknown parts of the maze.

The outline of this paper followed by the Introduction is related works, MARINS, method, result and discussion, the measurement of the maze, and conclusions.

## 2. Related Works

Augmented reality (or AR) technology and applications have long been investigated. In 1994, reality and virtuality were already considered as located at opposite ends of a continuum [1], and real and virtual objects in a real environment are to be combined, registered, and interacted in real-time [2]. The definition of AR was then gradually broadened to apply particular displays and sensors for auditory, tactile, and olfactory modalities [3]. Related systems have been utilized for different tasks for industrial equipment to lower error rates [4], for navigation systems in an art museum, a heritage site, or a theme park [5,6,7], for new medical applications in training, nursing, or operations [8,9,10], to stimulate pragmatic skills and general problem-solving abilities in preschoolers [11], to improve students’ perception of three projection planes of 3D shapes [12], and as an innovative pedagogical tool to deliver location-aware instructional materials from remote construction job sites to the classroom [13].

AR navigation has attracted significant attention from researchers in recent years. The development of a simple location-based AR system has constituted a main focus in indoor navigation. Tracking and positioning technologies have been created using different approaches. A mobile AR system was developed to guide a user traversing an unfamiliar building using a head-mounted display (HMD) and marker [14]. Marker detection experiments and case studies have been applied in facility management and have demonstrated feasibility and potential [15,16]. AR can also integrate with GPS [17,18,19], which is widely applied in outdoor AR navigation systems. However, its main disadvantage is that it has only a weak ability for indoor positioning due to inferior signal penetration [14,20,21,22]. Wi-Fi technology has also been applied [23,24]. However, signals tend to be extremely noisy and signal strength highly depends on surrounding building structures and materials [25]. Radio frequency identification (RFID) demands sufficient device setup and can be labor-intensive. On the other hand, researches have demonstrated the AR navigation system to be superior and possess great potential compared to using a traditional map [26,27,28].

Markers are frequently applied as a tracking and positioning aid in AR systems. Clear identification of a marker requires sufficient brightness in an environment to identify it and its context. Markers also need to be allocated within a defined range which is neither too far away for successful identification [15], nor too close to negatively impact environmental aesthetics [29]. Indeed, location-based AR has been shown to provide a better user experience than marker-based AR [30].

The mobile AR navigation system, which usually provides users with a convenient navigation experience, possesses great potential in integrating an environment for clear signage of paths without floating in the air [23,31] or preventing obstruction of real objects by larger arrow size [22,24,30]. AR users are likely to hesitate and return to the coming route once the instruction arrows are blocked by obstacles [17]. It is thus preferable to seamlessly integrate the AR system with a real environment to prevent misjudgments and provide path-switching options to assist users to find alternatives to reach the goal.

Very few studies mention the importance of lighting conditions in an environment. Most AR navigations are conducted in the daytime or with sufficient indoor brightness. The identification or recognition of markers also necessitates bright lighting to ensure a good tracking result [14,25]. However, in reality, dark situations occur frequently in disasters. Power failures are also common in such situations and can create a blackout condition with very limited available light sources. 

Wearable computing using Kinect for range data and feedback has been successfully developed for blind and visually impaired persons [32]. The system can be integrated with a path display function for additional indication of egress direction. Nevertheless, a popular smartphone-based AR can be more convenient for immediate responses to hazardous conditions. This study was specifically performed with a smartphone, especially an iPhone, for an easier SDK application development environment.

## 3. MARINS

**Figure 1:**
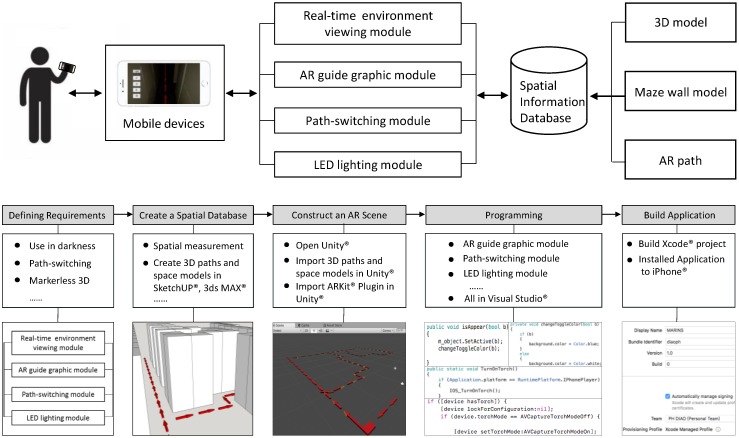
System structure (**top**) and development diagram (**bottom**) of MARINS.

**Figure 2:**
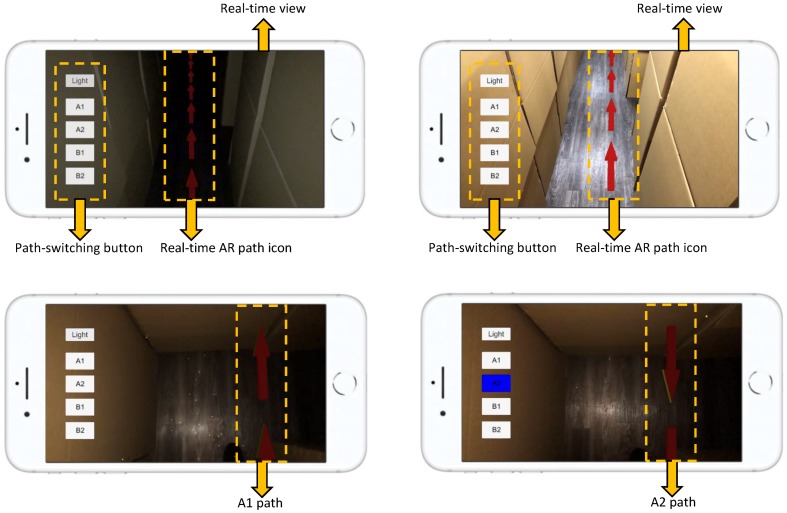
Screen shots of the MARINS interface in a dark environment (**top left**) and a bright environment (**top right**). The alternative path is selected by tapping the A2 option (**bottom**).

This study developed an iPhone-based AR system, i.e., MARINS, using Apple ARKit SDK on a Unity platform. The system consists of a real-time environment viewing module, an AR guide graphic module, a path-switching module, an LED lighting module, and a spatial information database (Figure 1). The modules interact with 3D spatial information through an iPhone 8 Plus screen to co-relate a subject location in a maze and to switch paths on-screen, as is needed for the shortest route to a goal. The physical setting and configuration of this maze are identical to the 3D computer model created in advance, in which the direction of the path to the goal is also modeled with explicit turns and arrows on the ground level. The MARINS interface is presented in Figure 2. The brightness of the red path, as a virtual object, is automatically adjusted as part of the function provided by ARKit Light Estimation. In other words, the path of red arrow looks brighter in a brighter environment lighting condition. Just like the screen of a smartphone, it has an option to automatically adjust the brightness based on the luminous level of ambient light.

The development diagram (Figure 1 bottom) starts from defining requirements, such as using the system in darkness, providing the path-switching option, and featuring markerless 3D tracking. Creating a spatial database includes the measurement and modeling of the 3D path and maze. Spatial database and ARKit plugin are imported into the Unity platform to build AR scenes. ARKit offers a version of SLAM technology. The original virtual cube intends to be moved sideways for frequent finger touches. This situation was modified by removing part of codes for a steadier display. After coding four modules and combining with iOS LED script and path-switching script, the application is created in a proj file to be installed on the iPhone.

MARINS possesses several useful functions for providing AR guidance of a route.

- This app is specifically tested for its level of accuracy to place the path within the maze in a relatively accurate manner to the wall and the goal with reference only made at the entrance.
- This app is useful for a dark environment of 0 lux, as measured in later tests, with the LED as the only light source.
- The app has a path-switching option that can be touched to show pre-installed and -modeled alternative routes to the goal.
- The app is also utilized as a video recording tool for the entire path traversed turn by turn.

**Figure 3:**
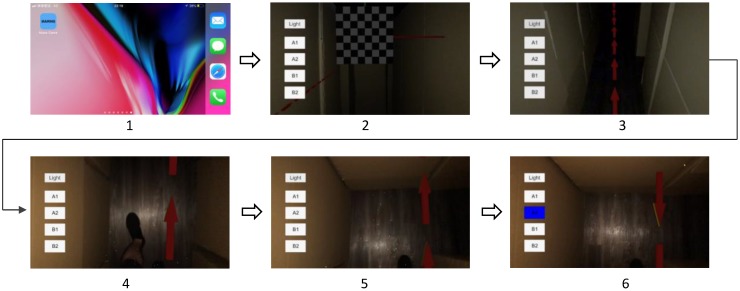
Operational procedures of MARINS.

A stepwise illustration of the operational procedures of MARINS is presented in Figure 3. (1) MARINS App icon; (2) alignment between the camera lens and the viewing angle by pointing the camera at the maze at the entrance; (3) the shortest path to Exit A appears (as shown in A1); (4) the subject moves by following the arrows; (5) route A1 is blocked; and (6) an alternative route is shown after the subject selects the A2 option. A video sample of the test can be seen in Appendix A.

## 4. Method

An experiment was performed in a university classroom to determine if the MARINS system reduces evacuation time and traveled distance, and leads to better acceptance.

### 4.1. Experiment Design of Test Space

**Figure 4:**
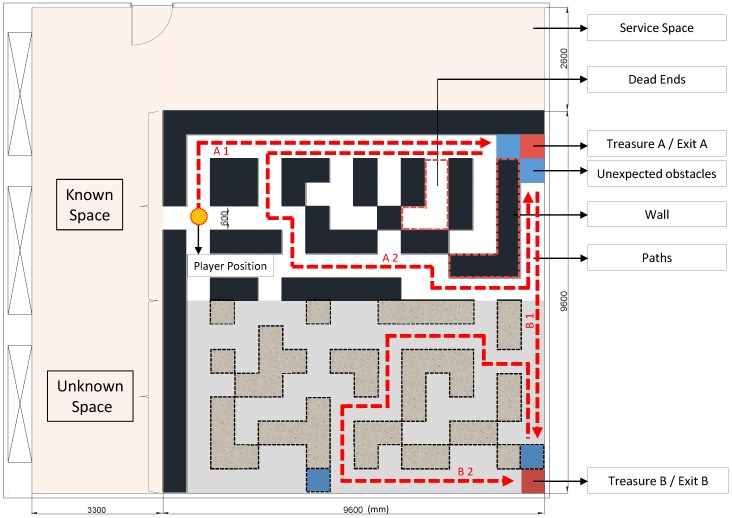
Maze layout (mm).

**Figure 5:**
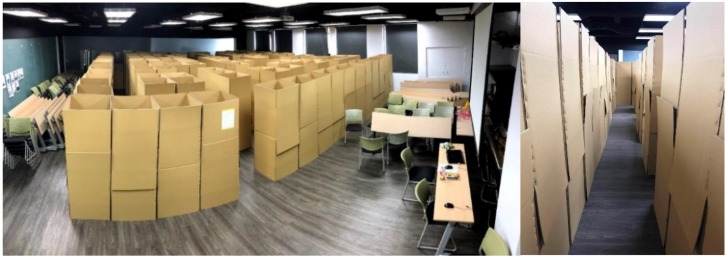
Panoramic view (**left**) and scene inside of the maze (**right**).

A maze (Figure 4) setup is used to test subjects using a traditional 2D map or MARINS. A maze of 92.16 m^2^ was assembled with cartons in an interior space (Figure 5), in which lights can be controlled during the experiment. The wall and path are 60 cm thick and 60 cm wide. The wall is 180 cm tall, which is sufficiently high to block the visibility of potential adjacent routes from above or underneath. In order to simulate a complicated maze composition in a limited space, design elements, such as dead ends, 0-lux darkness, unmarked obstacles, and unknown spaces, were added (Table 1).

This maze replicates a real environment, in that most of the space is familiar, but part of the space is subject to alteration due to unanticipated occurrences, such as earthquakes or fires. Similar to the layout of an egress route shown in a typical building evacuation map with instructions, the map already indicates the shortest path and an alternative route to Exit A on the map posted at the entrance.

An unknown environment can be classified as no spatial data are provided, or difference exists between personal perception and memory for real turns or current position in the maze. This study was made mainly for building space, so floor plans can be used as maps. However, difficulty still exists in finding a way out or identifying one’s location in a map.

The MARINS is designed for a real environment, for the most important of all, in a full scale (i.e., 1:1 scale). Thus, the maze and the entire space have to be modeled in full scale. The unknown environment is the set up that the testing subject can see, but has no clue of what is hidden behind the walls or each path may lead to. Thus, the subject has to try possible paths, as those are unknown to them. This test was made in an environment in which part of the space can be referred to map. There is another part and purposely design blockage cannot be referred to or found on the map. 

**Figure 6:**
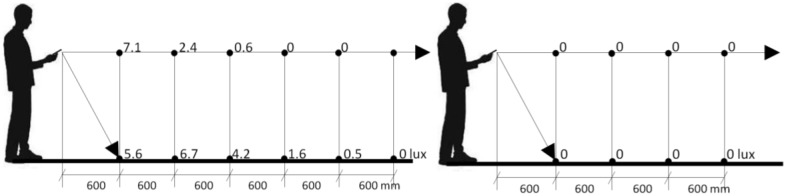
The interrelationship between lux and distance with light on (**left**) and off (**right**).

The test environment is in 0-lux darkness. The smartphone holding position is tilt forward to face ground level. The value of lux, in terms of distance, is shown in Figure 6, in which only approximately 3 m of distance ahead of the subject can be illuminated.

### 4.2. Experimental Rules

The test subjects have to use either MARINS or a 2D map to reach Exit A and Exit B. In order to reduce possible stress resultant from an evacuation or survival situation, this test is named the “Treasure Hunt Game”. All lights are off before the game begins. All of the tests were conducted in the evening to assure a 0-lux lighting condition. The treasure is located in Exit A and Exit B. The test subjects have to reach Exit B to achieve success. Exit A is located in a known space, and Exit B is located in an unknown space. In order to increase the challenge, Exit A is actually sealed, and the subjects have to search for Exit B instead. This is a one-person game with traversed time and distance recorded. This scenario also occurs in real life, in which a person could enter an unfamiliar environment searching for alternative egress routes after a fire exit is accidently blocked. Therefore, Exit A is closed before the game starts, while each test subject is unaware of this situation.

An assistant stood on a ladder with the viewing altitude high enough to see and mark down each subject’s movement indicated by the LED light. Since each unit is measured 60 × 60 cm, traveled distance is calculated by following the units each subject traveled. In addition, the recording function of mobile phone screen was turned on by the experimental group for additional verification. Another assistant was located at the exit to confirm each subject’s fulfillment of the test.

The rectangular maze is separated into two adjacent parts: The part that can be referred to with a 2D map is the known space, and the part that cannot be referred to is the unknown space. The subject is instructed to enter from the former part, encounter a few obstacles, and exit from the latter part to reach Exit B. While traversing from a relatively known terrain to an unknown one, each period of time is recorded separately.

As seen in the operational procedures (Figure 3), Exit A can be reached by route A1 and A2, and Exit B can be reached by route B1 and B2 (Figure 4). The interface provides another shortest route when the obstacle is encountered. The route is shown in red arrows. When an obstacle is encountered along A1, the option of A2 can be selected as an alternative to Exit A. If Exit A is blocked as well, B1 can be selected.

### 4.3. Subjects and Instruments

All subjects were briefed and were aware of the experimental content before they joined in the study. Test subjects were divided into an experimental group and a control group with approximately 15 each (Table 2). Thirty-one masters and Ph.D. students were randomly selected. The experimental group used an iPhone 8 Plus (iOS version 11.3) with MARINS. The control group used a traditional 2D map (Table 2), of which a picture can be taken with a smartphone. In the beginning, all test subjects were briefed about the location of the treasure, i.e., Exit A. While the subject is about to reach the first location, she or he will be informed that the treasure is gone and the second route for Exit B must be retrieved. The first and the second locations are located in the two parts, respectively.

The purpose of this AR study is to test the feasibility and satisfaction of using MARINS. The pathfinding time and traveled distance of the two groups are used as the validity measurement of assessment in time and distance, respectively. In the evaluation of satisfaction, the post-study system usability questionnaire (PSSUQ) was used in four dimensions, with a total of 16 items, including 1–16 items for the “overall average”, 1–6 items for “system usefulness”, 7–12 items for “information quality”, and 13–15 items for “interface quality.” The questionnaire items were scored on a Likert-type seven-point scale [33], where 1, 2, 3, 4, 5, 6, and 7 represented by “strongly agree”, “agree”, “somewhat agree”, “neutral”, “somewhat disagree”, “disagree”, and “strongly disagree”, respectively. The Cronbach’s α values of the four dimensions were 0.97, 0.97, 0.91, and 0.90, respectively, implying high reliability of the questionnaire.

The questionnaire is also feasible for a traditional egress plan in which some unsuitable or over-simplified situations can be removed. Twenty-three questions (see Appendix B) are designed, including basic information, such as gender, department, enrollment in drafting course, and familiarity with video games.

### 4.4. Experiment Procedure

**Figure 7:**
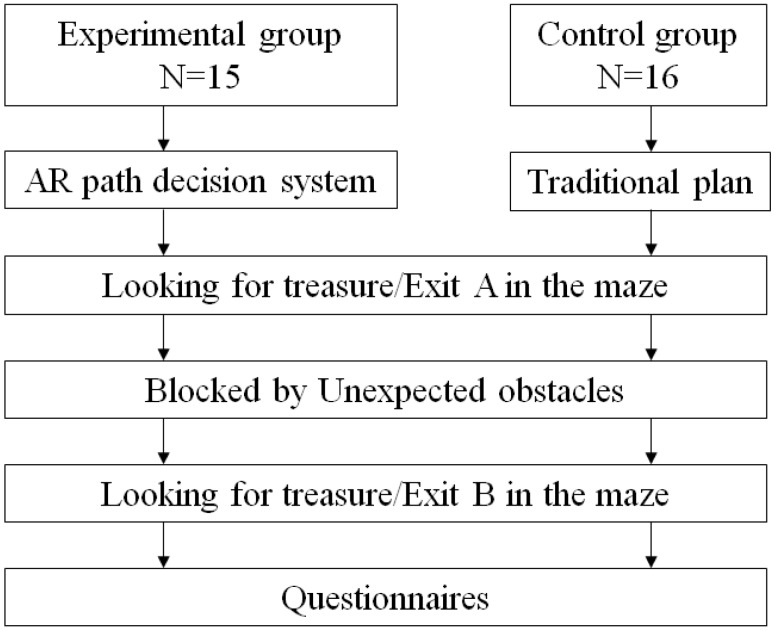
Experimental procedure.

As shown in Figure 7, the experimental group and the control group will first search for the treasure in Exit A using MARINS and a traditional 2D plan. Due to unexpected blockage of an obstacle, the original simple route is no longer available just prior to reaching Exit A. The subject then has to search for alternatives using the tool or information available until the second treasure at Exit B is reached. The investigator separates the time and distance of known space and unknown space. All subjects filled out the PSSUQ after reaching Exit B.

## 5. Result and Discussion

The test result shows difficulties in following the path in darkness for a longer detour. The complexity of the maze is discussed in terms of the measurement of the maze, after the analysis of pathfinding time, distance, and system usability.

### 5.1. Analysis of Pathfinding Time

Table 3 shows the t-test results of pathfinding time in total, known space, and unknown space of the two groups. It is found that the ratings of the experimental group were significantly lower than those of the control group in terms of total pathfinding time and known space pathfinding time with *t* = −2.65 (*p* < 0.05) and *t* = −3.00 (*p* < 0.001), respectively. The *d* values of the first two dimensions were 0.95 and 1.08, respectively, showing a large effect size [34]. The experimental group has a lower pathfinding time in unknown space than that of the control group, but the results are not very significant with *t* = −0.41 (*p* > 0.1). The *d* value of the unknown space was 0.15, revealing a smaller effect size. In other words, the MARINS system is shown to reduce pathfinding time in known space and in total summation compared to the application of a traditional 2D map. Unknown space is found to have little effect.

In Table 3, Table 4 and Table 5, *N* is the number of samples. SD is standard deviation; *T* is one of the values for identifying *p*-value. *P* is used to determine whether the data have a significant difference, while two sets of data are statistically significant when *p* < 0.05. *D* is the degree to which the null hypothesis is false, and the large effect size should be larger or equal to 0.8.

### 5.2. Analysis of Traveled Distance

Table 4 presents the t-test results of the traveled distance in total, known space, and unknown space of the two groups. It is found that the ratings of the experimental group were significantly lower than those of the control group in terms of traveled distance in total, known space, and unknown space with *t* = −8.42 (*p* < 0.001), *t* = −3.61 (*p* < 0.01), and *t* = −3.74 (*p* < 0.01), respectively. The *d* values of the three dimensions were 2.98, 1.28, and 1.32, respectively, showing a large effect size [34]. The results demonstrated that the MARINS system can reduce traveled distance compared to the application of a traditional 2D map.

### 5.3. Analysis of System Usability

The PSSUQ questionnaire was used with a seven-point Likert-type rating scale, in which 1 represents “highly agree” and 7 represents “highly disagree”. Essentially, the lower the rating, the higher the system’s usability. Table 5 shows the t-test results of the PSSUQ of the two groups. It is found that the ratings of the experimental group were significantly lower than those of the control group in terms of overall average, system usefulness, information quality, and interface quality with *t* = −5.01 (*p* < 0.001), *t* = −4.42 (*p* <0.001), *t* = −4.76 (*p* <0.001), and *t* = −4.86 (*p* <0.001), respectively. The *d* values of the four dimensions were 1.84, 1.56, 1.70, and 1.73, respectively, revealing a large effect size [34]. The MARINS system is shown to have significantly higher system usability than the application of a traditional 2D map.

## 6. The Measurement of the Maze

The measurement of the maze is used to explain the close match of time between the two groups in unknown spaces, in terms of maze size, number of dead ends, visibility, path length, and path configuration. Apparently, maze size increases the search space in terms of multiple layers of walls and paths. A path can be a dead end with either explicit or inexplicit visibility of the other end. A complicated dead end may also have walls entangled as traps. The knowledge of the map can be expanded as long as each traversed path is remembered. However, the lighting condition and the arrangement of dead ends, individually or in combination, significantly increases the difficulty of searching for the goal.

The first part of the maze, i.e., the known space, has a series of dead ends arranged and explored before a true egress can be found, even when a 2D map is provided. Consequently, the control group requires a longer time and distance for an alternative route to Exit A. The second part of the maze, i.e., the unknown space, has a mixed arrangement of dead ends and egress. Even when a map is not provided, searching for Exit B is relatively easier for test subjects in a small-scaled maze.

The two parts of the maze are timed separately as known space and unknown space.

- The timing in the known space is shorter with the assistance of AR. The measurement of this part of the maze was difficult for subjects even with the referred information of the map.
- The timing difference in the unknown space is smaller without the assistance of AR. The measurement of this part of the maze was easy for subjects even without referred information.
- The known space test showed that pathfinding is relatively difficult in a complicated environment, even if a reference to a map is provided.
- The unknown space test revealed that pathfinding is relatively easy in a simpler environment, irrespective of whether or not the AR tool is applied.

## 7. Discussion

The purpose of the present study is to test if a convenient AR system is available to suggest a useful path to exit when an obstacle is encountered and it is impossible to traverse it. In order to incorporate paths in real environments, AR is applied to show suggested paths with references to existing space. Based on related studies, reality can be classified into VR, AR, MR, or QR [35]. Thus, the AR specified in this study actually provides mediated information, such as the selection of paths. Thus, a maze was applied as a simplified environment, and a treasure hunt scenario was utilized for an experiment focused on pathfinding in darkness.

The detection of obstacles along a subject’s moving direction, while looking for an egress, constitutes two important tasks during evacuation. An effective combination of the two functions would be very beneficial. This study is aware of this aim, but focuses on the application for pathfinding, especially when a smartphone is considered as a target device to be applied. MARINS is considered to be an interior navigation system, and not a collision detection or warning system.

A MARINS user does not necessary always look at the screen or point to the ground. The smartphone body is slightly tilted towards the ground while a user can still look around him or herself to avoid possible hanging objects using the LED light. This constitutes a problem with great potential for harming people. So, instead of holding a map in one hand and a flashlight in the other, the light turns on automatically. Since the location of the path is anchored to the real space, a user can quickly go back to follow the path’s arrow after pointing the LED temporarily in another direction, such as where an overhanging obstruction is located.

Reality is different under various lighting conditions of brightness or entire darkness. It is also different for people who can see and for those born vision-impaired, since perception can differ markedly in linking the idea and configuration of an object. What would happen when AR is applied in darkness, especially with positioning-enabled from a distance? Should it be even more difficult to conduct indoor positioning in darkness? If a space is entirely dark, can any kind of AR be applicable, or become workable with the least modification? When we determined that AR can be applied using a smartphone in a bright space, but not in darkness, a first trial was performed to test if off-the-shelf night vision goggles can be attached for the same AR work. The answer was negative. However, whether we have to use a flashlight, or what level of lumens is requisite, for the same effect has yet to be determined. The most direct way is to use the LED light that comes with a smartphone and still provides sufficient brightness in a 0-lux situation for AR work.

Light is important in various types of hazards. Indeed, obstructions frequently occur during earthquakes or fires. This destruction presents a dynamic situation, in which an environment can continue to be demolished in different stages and in parts. Evacuation time is critical in saving lives. Many related studies have been conducted focusing on material, zoning, circulation, structure, and group evacuations. Numerous issues are involved in evacuations, such as fail-free circulation design, signage, and emergency lighting systems. These issues comprise different safety concerns under various types of hazards. However, searching for an egress route is always important. Flashlights are one of the most essential components in an emergency kit. This highlights the difficulty of evacuation in darkness. As long as sufficient brightness and head protection are available, obstructions can be seen and avoided with the least damage to the human body.

## 8. Conclusions

The MARINS system can reduce pathfinding time in known space and in total summation compared to the application of a traditional map or plan. It also reduces the traveled distance and achieves higher system usability in an unknown space. Moreover, a link can be created between the current location and the next orientation in darkness for people who are uncertain of the exact location of exits or turns. After positioning or orienting the paths correctly at the entrance, the arrows can lead subjects to the exit without noticeable tolerance, in a maze of the size 9.6 × 9.6 m. The indoor positioning during the test was able to assist subjects to refer oneself with the real maze walls and the locations of the path made by red arrows. While the maze design may lead to different perceptions in real world situations, a maze does constitute an approximation of a real environment under different complexity. The result enables the discovery of more potential types of maze measurement in a space.

Currently, all building spaces should be well-defined to serve building information modeling (BIM) needs. With advanced 3D modeling and Augmented Reality technology, a relatively complicated indoor space can be traveled with the assistance of a smartphone without GPS functionality. Most importantly, the present study confirms that the MARINS is feasible for evacuations in the most challenging conditions of darkness. The real evacuation time may vary, however, with the preloaded suggestions of shortest paths at each turning point, and users can be provided with more explicit choices to successfully cope with unexpected occurrences. While the location of turning points and the selection of alternatives deserve more thorough planning and additional 3D modeling efforts, the physical setting can be tested statistically in real sites in order to determine system effectiveness and efficiency. MARINS constitutes a useable application and evaluation tool for indoor navigation.

Future tests can involve multiple subjects for group simultaneous navigation and evacuation. Alternative paths can also be increased in spatial databases and provided to participants for identifying the shortest path to goal. The experimental site can also be modified with various settings of wall thickness, complexity, and path length, in order to elucidate the system’s usability in different situations.

## Figures and Tables

**Table 1:** Maze elements and related influence.

Element	Influence
Dead ends	Each path segment can be either a dead end or a route to another space, such as a living room, kitchen, or conference room.
Brightness	Brightness has a strong influence on a person’s awareness of location. As power failure frequently occurs in a disaster, a space can be totally dark or partially illuminated by an emergency lighting system.
Unexpected obstacles	Falling ceilings or broken construction elements can occasionally prevent people’s evacuation from a path with which they used to be familiar. So, unexpected instances of obstacles are installed without any indication on the map.
Unknown space	In reality, spaces are divided into known space, with which a person is familiar, and unknown space, which is new to a person or the original configuration is changed dramatically and no match can be found. It has become impossible for a test subject to search for another path to reach Exit A. As the subject is traversing from a known part of the maze to an unknown part, the location of Exit B is also unknown.

**Table 2:** Test groups and rules.

Group	*N*	Tool	Rules	Tool Icon
Experimental 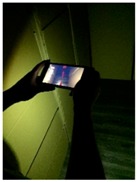	16	MARINS	Subject uses MARINS and smartphone light as the only source of light.	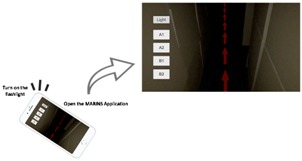
Control 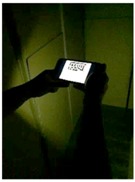	15	2D map	Subject can take a picture of the map as a reference. Subject uses smartphone light as the only source of light.	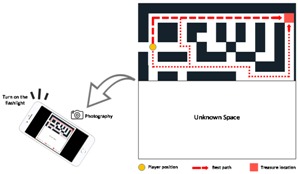

**Table 3:** Independent sample *t*-test of path-finding time between the two groups.

	Group	*N*	Mean(s)	SD	*t*	*d*
Total pathfinding time	Experimental	15	81.71	19.39	−2.65 *	0.95
	Control	16	101.44	21.89		
Known space pathfinding time	Experimental	15	47.50	15.50	−3.00 **	1.08
	Control	16	66.05	18.64		
Unknown space pathfinding time	Experimental	15	34.21	4.82	−0.41	0.15
	Control	16	35.39	10.19		

* *p* < 0.05, ** *p* < 0.01.

**Table 4:** Independent sample *t*-test of traveled distance between the two groups.

	Group	*N*	Mean(m)	SD	*t*	*d*
Total pathfinding distance	Experimental	15	49.84	1.48	−8.42 ***	2.98
	Control	16	66.68	7.85		
Known space pathfinding distance	Experimental	15	27.60	0.88	−3.61 **	1.28
	Control	16	35.03	8.18		
Unknown space pathfinding distance	Experimental	15	22.24	0.89	−3.75 **	1.33
	Control	16	31.65	10.00		

** *p* < 0.01, *** *p* < 0.001.

**Table 5:** Independent sample *t*-test of PSSUQ between the two groups.

	Group	*N*	Mean	SD	*t*	*d*
Overall average	Experimental	15	1.79	0.54	−5.10 ***	1.85
	Control	16	3.61	1.28		
System usefulness	Experimental	15	1.66	0.53	−4.42 ***	1.83
	Control	16	3.36	1.45		
Information quality	Experimental	15	1.83	0.66	−4.76 ***	1.70
	Control	16	3.61	1.33		
Interface quality	Experimental	15	2.04	0.68	−4.86 ***	1.73
	Control	16	3.98	1.43		

*** *p* < 0.001.

## Appendix A. Video Sample of the Test

https://youtu.be/HHb1kJAeJUw.

## Appendix B. Questionnaires

**Set 1: User information**

1	Age
2	Gender
3	Department
4	Have you enrolled in drafting course?
5	Have you ever played an AR game?
6	Have you ever played a sports game?
7	Have you ever played a real maze game?

**Set 2: PSSUQ**

1	Overall, I am satisfied with how easy it is to use this system.
2	It was simple to use this system.
3	I was able to complete the tasks and scenarios quickly using this system.
4	I felt comfortable using this system.
5	It was easy to learn to use this system.
6	I believe I could become productive quickly using this system.
7	The system gave error messages that clearly told me how to fix problems.
8	Whenever I made a mistake using the system, I could recover easily and quickly.
9	The information (such as buttons, on-screen messages, and path instructions) provided with this system was clear.
10	It was easy to find the information I needed.
11	The information was effective in helping me complete the tasks and scenarios.
12	The organization of information on the system screens was clear.
13	The interface of this system was comfortable.
14	I liked using the interface of this system.
15	This system has all the functions and capabilities I expect it to have.
16	Overall, I am satisfied with this system.
